# Chronic Guillain-Barré syndrome and refractory diarrhea after RYGB: a 9-year longitudinal case report

**DOI:** 10.1093/jscr/rjaf596

**Published:** 2025-08-13

**Authors:** Liu Yanzhang, Zhao Xiangwen, Wang Hui, Ji Jinfeng, Shi Shengli

**Affiliations:** Department of Bariatric and Metabolic Surgery, Xiaolan People’s Hospital of Zhongshan, No. 65, Jucheng Avenue, Xiaolan Town, Zhongshan, Guangdong 528415, China; Department of Bariatric and Metabolic Surgery, Xiaolan People’s Hospital of Zhongshan, No. 65, Jucheng Avenue, Xiaolan Town, Zhongshan, Guangdong 528415, China; Department of Neurology, Xiaolan People’s Hospital of Zhongshan, No. 65, Jucheng Avenue, Xiaolan Town, Zhongshan, Guangdong 528415, China; Department of Nutrition and Dietary Guidance, Xiaolan People’s Hospital of Zhongshan, No. 65, Jucheng Avenue, Xiaolan Town, Zhongshan, Guangdong 528415, China; Department of Gastroenterology, Xiaolan People’s Hospital of Zhongshan, No. 65, Jucheng Avenue, Xiaolan Town, Zhongshan, Guangdong 528415, China

**Keywords:** Guillain-Barré syndrome, diarrhea, type 2 diabetes, RYGB, bariatric surgery

## Abstract

We present a 46-year-old male who developed chronic Guillain-Barré syndrome and refractory diarrhea after undergoing Roux-en-Y gastric bypass, with progressive neurological deterioration and 34.9% total weight loss over 9 years. Serial evaluations demonstrated severe deficiencies include vitamin D, calcium, iron and other nutritions, accompanied by evidence of autoimmune dysregulation. The patient’s condition progressed to require partial parenteral nutrition due to worsening neurological and gastrointestinal dysfunction. This case highlights the critical need for ongoing nutritional monitoring after bariatric surgery and suggests that persistent micronutrient deficiencies may contribute to sustained immune dysfunction, potentially triggering chronic autoimmune neurological disorders in susceptible individuals.

## Introduction

Guillain-Barré syndrome (GBS) is classically an acute, immune-mediated polyneuropathy, with chronic forms account for less than 5% of cases [1–3]. Roux-en-Y gastric bypass (RYGB), while effective for obesity management, carries lifelong risks of micronutrient malabsorption and refractory gastrointestinal complications, including chronic diarrhea [4, 5]. While vitamin deficiencies post-RYGB are well-documented, However, their role in triggering chronic autoimmune neuropathy is poorly understood.

## Case report

A 46-year-old male with a 6-year history of poorly controlled type 2 diabetes (HbA1c 9.8%, BMI 30.3 kg/m^2^). The patient presented with severe metabolic dysregulation (random glucose 6–25 mmol/L, predominantly 16–25 mmol/L) due to no adherence with metformin, glipizide, and long-acting insulin, resulting in bilateral diabetic retinopathy (left eye with 6-month blurred vision) and nephropathy (elevated urinary albumin despite preserved glomerular filtration rate), demonstrating progressive microvascular complications from sustained hyperglycemia.

### Operation and postoperative course

Laparoscopic RYGB was performed, creating a 30 mL gastric pouch with a 100 cm Roux limb measured from the ligament of Treitz. The patient recovered well, with stable glucose (6.6–9.8 mmol/L) and transient low-grade fever (peak 38.3°C) that resolved spontaneously, By postoperative day 8 (POD 8) he achieved a 4 kg weight loss and was discharged on a semi-liquid diet. At POD 21, progressive neurological deficits emerged: bilateral lower limb weakness (distal 3/5, proximal 3–4/5 Medical Research Council (MRC) grade), distal numbness (feet-predominant), with mild hyperalgesia and dorsal hypoesthesia; urinary retention with suprapubic discomfort; and systemic symptoms (dizziness, anorexia) without febrile or focal neurological deficits. Neurological examination revealed diminished lower limb reflexes, preserved upper limb reflexes, bilateral Lasègue’s sign positivity, and absent meningeal signs, while maintained normal bowel function.

### Diagnostic and therapeutic course

The patient showed albuminocytologic dissociation in cerebrospinal fluid, elevated protein 694.3 mg/L and cells 5/μL, and electrophysiological evidence of proximal nerve root involvement (abnormal F-waves and H-reflexes), meeting Brighton criteria for GBS diagnosis. Intravenous immunoglobulin (IVIG) administration after definitive diagnosis, partial neurological recovery was achieved, with distal lower limb strength improving to MRC grade 4/5 by postoperative day 48 and achieved functional independence in ambulation independence. However, the patient subsequently developed gradual-onset diarrhea (1–2 episodes/day, Bristol Type 5–6).

### 9-year follow-up

The patient received ongoing dietary counseling focusing on nutrition optimization, micronutrient supplementation, and lifestyle adjustments. Despite strict dietary compliance, persistent diarrhea and inadequate intake led to unresolved micronutrient deficiencies. The patient reported chronic diarrhea with 4–8 daily episodes, occasionally exceeding 10. Stools were consistently pasty (Bristol stool scale Type 5–6) without abdominal pain or systemic symptoms. Although combination anti-diarrheal therapy provided partial relief, symptoms often recurred, significantly impacting daily life. Persistent left lower limb weakness resulted in a mild limping gait. While maintaining independent ambulation, they required minimal activities of daily living assistance and reported prolonged squatting difficulty. These residual symptoms did not significantly impair basic self-care. 

In 2022 (postoperative year 6), the patient was hospitalized for severe diarrhea evaluation. The patient demonstrated mild distal-predominant weakness (lower limbs 3–4/5 distally and 4/5 proximally, upper limbs uniformly 4/5) with preserved muscle tone but mild atrophy, accompanied by distal lower limb hyperalgesia and dorsal foot hypoesthesia with intact proprioception/vibration. Reflexes showed diminished patellar and Achilles responses with flexor plantar reflexes, while special tests revealed bilateral Lasègue’s sign positivity. Electromyography identified reduced motor conduction velocity in the left tibial nerve (normal latency/amplitude), normal motor and sensory conduction velocities with decreased sensory amplitudes in the right tibial and bilateral median/ulnar/radial nerves, and prolonged F-wave latency (normal occurrence rate) in the right tibial nerve, consistent with sensory fiber impairment affecting all limbs with right tibial F-wave abnormality.

### Clinical summary (2016–2025)

The patient developed progressive weight reduction (BMI 19.7 kg/m^2^, 34.9% weight loss) with persistent muscle weakness, refractory diarrhea (4–8 daily Bristol type 6–7), and asymmetric neuropathic weakness, resulting in dependence and work disability. Secondary complications included moderate depression/anxiety (Patient Health Questionnaire-9: 18) exacerbated by medication non-adherence. Further details are provided in (Table 1).

**Table 1:** Clinical and biological parameters before and at 6^th^ year after operation

	At baseline	At 6^th^ year after operation	Reference range^c^
Clinical parameters			
Weight,kg	87.6	56.9	
BMI,kg/m2	30.3	19.7	
Weight loss, kg(%)		30.6(34.9)	
Upper limbs(MRC grade)		4/5	
Lower limbs (MRC grade)		3–4/5	
Biological parameters			
Iron (μmol/L)	14.28	4.77^a^	7.2–27.7
Folate (nmol/L)	19.6	43.6^b^	8.8–39.7
Ferritin (ng/mL)	942.8^b^	273.9	30–400
Vitamin B12 (pmol/L)	1476^b^	701.7^b^	148–660
Total 25-OH Vitamin D (nmol/L)	NM	32.7^a^	>75
Calcium (mmol/L)	2.10	1.78^a^	2.08–2.6
Albumin (g/L)	48.1	29.4^a^	40–55
Prealbumin (mg/L)	234	133^a^	200–400
RBC (×10^12^/L)	4.89	3.30^a^	4.3–5.8
Hemoglobin (g/L)	147	95^a^	130–175
Blood Glucose (mmol/L)	6.8–22.8 (72% >13)	5.7–20.3 (70% >13)	3.9–6.1
HbA1c (%)	8.7^b^	5.5	4–6
TSH (μIU/mL)	1.460	0.997	0.27–4.2
Total T4 (nmol/L)	78.45	44.72^a^	66–181
Stool Fungal Test	Negative	*Saccharomyces cerevisiae*	Negative

NM: not measured; RBC: red blood cell; TSH: thyroid-stimulating hormone.

^a^Below reference range.

^b^Above reference range.

^c^Reference ranges provided by the clinical laboratory of Xiaolan People’s Hospital of Zhongshan.

Clinical parameters include weight, BMI, weight loss percentage, and muscle strength (MRC grading).

Biological parameters cover iron, folate, vitamin levels, blood counts, glucose, thyroid function, and stool fungal test results.

Upper/lower limb muscle strength graded on the Medical Research Council (MRC) scale (0–5).

Blood glucose and HbA1c trends indicate persistent dysregulation post-operation.

Longitudinal analysis reveals significant metabolic and nutritional alterations over the six-year interval. Marked deficiencies developed in iron metabolism and hematopoietic parameters, accompanied by paradoxical vitamin fluctuations (elevated folate with declining B12). While glycemic control demonstrated substantial improvement, persistent dysregulation was evident through continued hyperglycemic episodes. The metabolic profile shows partial correction of insulin resistance alongside emerging hypocalcemia. Notably, new microbiological findings were identified and hypothyroidism. These findings highlight progressive micronutrient depletion coexisting with partially resolved metabolic dysregulation, suggesting complex pathophysiology requiring multidimensional interpretation.

## Discussion

Bariatric surgery has become an important treatment for obesity and T2DM. Significant neurological complications have been reported [6, 7], including postoperative complications range from common manifestations like peripheral neuropathy (60%) and encephalopathy (30%) to rare but severe conditions such as GBS [8–11]. The pathophysiology remains unclear but appears driven by nutrient deficiencies (particularly thiamine [B1] and cobalamin [B12]) that likely trigger immune dysfunction and demyelination [12, 13]. This case demonstrates the occurrence of acute GBS following RYGB. Although IVIG therapy led to initial improvement, the condition progressed into chronic GBS accompanied by refractory diarrhea, suggesting that this persistent diarrhea likely induced malabsorption of multiple nutrients [5, 14]. This highlights the critical need for postoperative surveillance of GBS following bariatric procedures, with particular attention to its potential association with altered immune homeostasis and gut microbiome dysbiosis. These findings hold significant implications for optimizing patient management through prophylactic micronutrient supplementation and structured neurological monitoring protocols in postoperative care [13].

## Data Availability

The data supporting the findings of this article are available within the text.

## References

[1] Van den Bergh P, van Doorn P, Hadden R, et al. European academy of neurology/peripheral nerve society guideline on diagnosis and treatment of chronic inflammatory demyelinating polyradiculoneuropathy: report of a joint task force-second revision. Eur J Neurol 2021;28:3556–83. 10.1111/ene.14959 34327760

[2] Doets AY, Verboon C, van den Berg B, et al. Regional variation of Guillain-Barré syndrome. Brain 2018;141:2866–77. 10.1093/brain/awy232 30247567

[3] Bellanti R, Rinaldi S. Guillain-Barré syndrome: a comprehensive review. Eur J Neurol 2024;31:16365. 10.1111/ene.16365 PMC11235944 38813755

[4] Gasmi A, Bjørklund G, Mujawdiya P, et al. Micronutrients deficiences in patients after bariatric surgery. Eur J Nutr 2022;61:55–67. 10.1007/s00394-021-02619-8 34302218

[5] Borbély YM, Osterwalder A, Kröll D, et al. Diarrhea after bariatric procedures: diagnosis and therapy. World J Gastroenterol 2017;23:4689–700. 10.3748/wjg.v23.i26.4689 28765690 PMC5514634

[6] Bodunova N, Degterev D, Suponeva N, et al. Guillain-Barré-like syndrome after bariatric surgery. Bariatric Times 2015;12:16–8.

[7] Berger JR, Singhal D. The neurologic complications of bariatric surgery. Handb Clin Neurol 2014;120:587–94. 10.1016/B978-0-7020-4087-0.00039-5 24365339

[8] Hamdeh MA, Mounshar A, Asad RM, et al. Guillain-Barré syndrome following laparoscopic sleeve gastrectomy: a tale of two cases. Obes Surg 2025;35:345–9. 10.1007/s11695-024-07635-1 39680291

[9] Wu Q, Li FY, Hu J, et al. Guillain-Barré syndrome following weight loss: a review of five diet-induced cases and nineteen bariatric surgery cases. Front Neurol 2025;16:1557515. 10.3389/fneur.2025.1557515 40201020 PMC11975578

[10] Li X, Zhang C. Guillain-Barré syndrome after surgery: a literature review. Front Neurol 2024;15:1368706. 10.3389/fneur.2024.1368706 PMC11024248 38638310

[11] Ishaque N, Khealani BA, Shariff AH, et al. Guillain-Barré syndrome (demyelinating) six weeks after bariatric surgery: a case report and literature review. Obes Res Clin Pract 2015;9:416–9. 10.1016/j.orcp.2015.02.001 25765350

[12] Bahardoust M, Eghbali F, Shahmiri SS, et al. B1 vitamin deficiency after bariatric surgery, prevalence, and symptoms: a systematic review and meta-analysis. Obes Surg 2022;32:3104–12. 10.1007/s11695-022-06178-7 35776243

[13] Parrott J, Frank L, Rabena R, et al. American Society for Metabolic and Bariatric Surgery Integrated Health Nutritional Guidelines for the surgical weight loss patient 2016 update: micronutrients. Surg Obes Relat Dis 2017;13:727–41. 10.1016/j.soard.2016.12.018 28392254

[14] Mechanick JI, Apovian C, Brethauer S, et al. Clinical practice guidelines for the perioperative nutrition, metabolic, and nonsurgical support of patients undergoing bariatric procedures - 2019 update: cosponsored by American Association of Clinical Endocrinologists/American College of Endocrinology, the Obesity Society, American Society for Metabolic and Bariatric Surgery, Obesity Medicine Association, and American Society of Anesthesiologists. Endocr Pract 2019;25:1346–59. 10.4158/GL-2019-0406 31682518

